# Communities conquering COVID-19: Black and Latinx community perspectives on the impact of COVID-19 in regions of Michigan hardest hit by the pandemic

**DOI:** 10.1017/cts.2024.591

**Published:** 2024-12-03

**Authors:** Ayse G. Buyuktur, Fernanda L. Cross, Jodyn Platt, Jasmin Aramburu, Pranati Movva, Ziyu Zhao, Tiffany Cornwall, Rebecca Hunt, Jo Ann McCollum, Angela Reyes, Charles E. Williams, Arthi Ramakrishnan, Barbara Israel, Erica E. Marsh, Susan J. Woolford

**Affiliations:** 1 Michigan Institute for Clinical and Health Research, University of Michigan, Ann Arbor, USA; 2 School of Social Work, University of Michigan, Ann Arbor, USA; 3 Department of Learning Health Sciences, University of Michigan Medical School, Ann Arbor, USA; 4 College of Osteopathic Medicine, Michigan State University, East Lansing, USA; 5 Department of Obstetrics and Gynecology, University of Michigan Medical School, Ann Arbor, USA; 6 Department of Health Management and Policy, School of Public Health, University of Michigan, Ann Arbor, USA; 7 New West Willow Neighborhood Association, Ypsilanti, USA; 8 Detroit Hispanic Development Corporation, Detroit, USA; 9 Department of Health Behavior and Health Education, School of Public Health, University of Michigan, Ann Arbor, USA; 10 Susan B Meister Child Health Evaluation and Research Center, Department of Pediatrics, University of Michigan, Ann Arbor, USA

**Keywords:** Impact of COVID-19, qualitative research, disparities, social determinants of health, communities of color

## Abstract

**Introduction:**

In Michigan, the COVID-19 pandemic severely impacted Black and Latinx communities. These communities experienced higher rates of exposure, hospitalizations, and deaths compared to Whites. We examine the impact of the pandemic and reasons for the higher burden on communities of color from the perspectives of Black and Latinx community members across four Michigan counties and discuss recommendations to better prepare for future public health emergencies.

**Methods:**

Using a community-based participatory research approach, we conducted semi-structured interviews (*n* = 40) with Black and Latinx individuals across the four counties. Interviews focused on knowledge related to the pandemic, the impact of the pandemic on their lives, sources of information, attitudes toward vaccination and participation in vaccine trials, and perspectives on the pandemic’s higher impact on communities of color.

**Results:**

Participants reported overwhelming effects of the pandemic in terms of worsened physical and mental health, financial difficulties, and lifestyle changes. They also reported some unexpected positive effects. They expressed awareness of the disproportionate burden among Black and Latinx populations and attributed this to a wide range of disparities in Social Determinants of Health. These included racism and systemic inequities, lack of access to information and language support, cultural practices, medical mistrust, and varied individual responses to the pandemic.

**Conclusion:**

Examining perspectives and experiences of those most impacted by the pandemic is essential for preparing for and effectively responding to public health emergencies in the future. Public health messaging and crisis response strategies must acknowledge the concerns and cultural needs of underrepresented populations.

## Introduction

In the United States (U.S.), racial and ethnic disparities associated with COVID-19 are well documented [1]. In peak periods of the pandemic, infection, hospitalization, and death rates from COVID-19 were higher for Black and Latinx populations compared to Whites [1]. There is also emerging evidence that Black and Latinx individuals have a higher likelihood of experiencing physical, cognitive, or mental health conditions related to long COVID, indicating ongoing racial and ethnic health disparities related to the pandemic [2,3].

Studies show that COVID-19’s disproportionate impact on Black and Latinx populations is likely due to disparities in access to healthcare and a higher prevalence of comorbidities [4–6]. In addition, systemic biases and racism put these communities at higher risk of exposure to the virus [7,8]. Contributing factors include residential segregation, overcrowded living conditions, and wealth inequality, all of which lead to poorer health outcomes [9–11]. Additionally, Black and Latinx people are more likely to have jobs that cannot be performed remotely and they are more likely to use public transportation, making it more difficult to follow guidelines for avoiding infection [12,13]. Risk of deportation and family separation is another important social determinant of health for immigrant populations as it compounds individuals’ stressors, increasing their health risks [14]. All these inequities are deeply rooted in historical and ongoing social, economic, and cultural injustices that propagate institutional mistrust among members of these communities [15].

General medical mistrust has fueled vaccine and treatment hesitancy regarding COVID-19 in the United States [16]. Public health responses to the pandemic exist in the context of centuries of systemic racism and mistrust. In Black and Latinx communities, past experiences with racism, exemplified by experimentation on enslaved Black women without anesthesia, the exploitation of Henrietta Lacks’s cells, and the Puerto Rico Pill Trials, contribute to distrust of the medical system and scientific communities [17–19]. This mistrust extends to public health responses as a whole, including (but not limited to) government messaging and vaccination campaigns among Black and Latinx communities [20–22]. To further compound the issue, misinformation about COVID-19 disseminated via television, the internet, and social media has made it difficult for people to identify which sources of information to trust, hampering the ability of public health messages to positively impact behavioral choices among many populations [23,24].

Addressing these inequities requires creating space for honest discussions to be able to understand the lived experiences of racial and ethnic minority communities. Within the first year of the pandemic, the National Institutes of Health (NIH) created the Community Engagement Alliance (CEAL) against COVID-19 Disparities to engage with and learn from severely impacted communities [25]. This paper reports findings from Michigan CEAL’s work in four Michigan counties during a peak pandemic period in 2020–2021. During this time, the impact of COVID-19 on Black and Latinx communities in Michigan was significant. The disparities between Black Michigan residents and their White peers were particularly pronounced; mortality from COVID-19 was disproportionately higher even after accounting for demographic and underlying health characteristics [26,27]. The cumulative rate of COVID-19 cases in Black populations was over 40% higher than among White populations [28]. Simultaneously, Latinx Michigan residents also experienced greater burdens than non-Latinx residents, with death rates nearly double their non-Latinx counterparts [28].

Besides the disproportionately high rates of COVID-19 infection and deaths, the unemployment rate was 35.5% for Black Michigan residents, as compared to 17.5% for White Michigan residents, and Black residents experienced unprecedented rates of housing insecurity at this time [28]. Additionally, both Black and Latinx Michigan residents were more likely to be in vulnerable situations serving as essential workers and otherwise at higher risk of contracting the virus [28].

The goal of this study was to examine the impact of COVID-19 on Black and Latinx communities during a peak pandemic period, and to share in their own words their perspectives about the factors that contributed to the higher impact. Furthermore, participants’ insights regarding actions that could improve outcomes for their communities were elicited. Understanding these experiences and perspectives is important for engaging with these communities in more culturally appropriate ways, and preparing for future public health emergencies.

## Materials and methods

We conducted in-depth interviews as part of the Michigan CEAL (MICEAL) initiative. MICEAL aims to support solutions to mitigate COVID-19-associated healthcare burdens in underrepresented communities. We interviewed Black and Latinx residents in four Michigan counties significantly impacted by the pandemic: Genesee (major city: Flint), Kent (major city: Grand Rapids), Washtenaw (major city: Ypsilanti), and Wayne County (major city: Detroit). Black individuals make up between 10% and 38% of county residents, while Latinx individuals make up between 4% and 11% of residents [29]. About 6% of Black Michigan residents and 12% of Latinxs are uninsured [29]. Michigan is home to 1.2% undocumented immigrants, about 52% of whom are of Latinx origin [31,32].

MICEAL is a community-academic partnership between University of Michigan researchers and a Steering Committee (SC), which at the time of data collection included 16 community leaders from 15 community-based organizations and health and human service agencies across these counties, as well as 3 academic members. Using a community-based participatory research (CBPR) approach, the study team collaborated with the SC to develop research questions, an interview protocol, an informed consent document, recruitment strategies, and data interpretation [33]. Throughout implementation, the study team participated in the monthly SC meetings, providing updates and holding group discussions on emerging findings. This study was approved by the University of Michigan’s Institutional Review Board.

### Data collection

From December 2020 through June 2021, we conducted 40 virtual semi-structured interviews with Black (*n* = 24) and Latinx (*n* = 16) residents. By design, 10 participants were recruited in each county. For recruitment, we distributed study flyers in English and Spanish with the help of Steering Committee members. Community members 18 years of age or older who self-identified as Black and/or Latinx were eligible to participate. We conducted the interviews in the language of the participants’ choosing (English or Spanish). Each participant received a $50 gift card for participation.

Interview questions and prompts reflected five priority areas provided to CEAL teams across the nation by the NIH at the time: (1) fundamental knowledge of the pandemic; (2) impact on communities; (3) sources of information; (4) testing and diagnosis; and (5) attitudes toward vaccination and participation in vaccine trials. We also collected demographic information.

All interviews lasted between 45–60 minutes and were conducted via the Zoom video conferencing platform. A copy of the informed consent document was sent to each participant prior to the interview, and a study team member reviewed it with them via phone or video conference. Participant oral consent was documented on the consent form. We recorded and professionally transcribed all interviews except for one (upon participant request); a study team member took detailed notes during the unrecorded interview. Spanish-language transcripts were translated into English by professional translators. Bilingual members of the research team reviewed all transcripts for accuracy and removed individually identifying information.

### Data analysis

The study team conducted a qualitative analysis of the interview transcripts by iterative inductive coding and identified key themes highlighted in the interviews. First, three pairs of researchers analyzed the same four transcripts. All pairs then came together for full-team discussions to develop a thematic codebook. The team then used four additional transcripts to calculate inter-rater reliability (IRR = 0.81). The IRR was computed in Dedoose Version 9.0.17, a web application for managing, analyzing, and presenting qualitative and mixed-method research data. As appropriate, new themes were added to the codebook following team discussion. The study team coded all transcripts using a final codebook. At weekly meetings, the study team discussed and summarized emerging findings until data saturation was observed. All coders had previous experience with qualitative data analysis. To ensure adequate training, all coders reviewed the interview guide, the data, and the process for using the analysis software with senior study team members. Monthly discussions with the SC included data interpretation and ideation for how study findings might be translated into recommendations for action.

## Results

Table 1 shows the distribution of interviews across counties and participant demographics. Most participants were women (80%). Younger adults were underrepresented. The educational level of participants was greater than the national average, with most participants indicating they had at least some college education. Participants reported a diverse range of professions including healthcare, frontline workers in maintenance, retail, production, hospitality, banking, and education. A portion of the study’s participants were either retired or semi-retired, and one was on disability.

**Table 1.** Participant demographics by county

	Genesee	Kent	Washtenaw	Wayne	Full Sample
County	*n*	*n*	*n*	*n*	%
Race/Ethnicity					
Latinx	4	4	4	4	40%
Black	6	6	6	6	60%
Age					
18–29	1	0	1	0	5%
30–59	6	8	6	8	70%
60+	3	2	3	2	25%
Gender					
Woman	8	7	10	7	80%
Man	2	3	0	3	20%
Education					
Less than a High School Diploma	1	1	0	1	<10%
High School Diploma or Equivalent	0	2	1	3	15%
Some College	4	4	2	4	35%
College Degree	1	3	6	0	25%
Higher than a College Degree	4	0	1	1	15%
Unknown	0	0	0	1	<10%
Average Number of People per Household	2.9	3.1	2.8	2.77*	

*Note. N* = 40 (*n* = 10 for all but 1 condition). *n = 9. There is missing data for one participant.

Our findings are organized under three overarching themes: (1) impact of COVID-19 on individuals; (2) perspectives on why the pandemic’s impact has been higher on Black and Latinx communities; and (3) strategies used to alleviate the impact of COVID-19. Each of these themes yielded multiple sub-themes. We explore these findings with exemplary quotes from each sub-theme.

## Impact of COVID-19 On Individuals

Participants commonly described the impact of the pandemic as “significant.” The examples they provided highlight five sub-themes: (1) worsened physical health; (2) worsened mental health; (3) financial difficulties; (4) unwanted changes in lifestyles; and (5) positive outcomes.

### Worsened physical health

Participants described experiencing various symptoms of COVID-19, and talked about community members who suffered severely from infection by the virus:

And then I see people coming out of it who still need rehabilitation after six months. Still can’t fully walk, still can’t breathe. —*Black Woman*

Participants also reported a negative impact on their health from lifestyle changes and stress caused by the pandemic:

I gained weight, approximately 10 kilos, I had super high cholesterol, triglycerides… it is a decision that you say, “Well, again, I am going to continue living with this. I have to take measures based on my health because if COVID does not kill me, something else will kill me,” because anxiety and stress can generate a lot of things for you. —*Latinx Woman*

### Worsened mental health

For participants, possibly the greatest impact of the pandemic was on their mental health. Among the many descriptors used, the most common were: fear, stress, anxiety, grief, and trauma. Participants expressed fear of getting sick and spreading the virus to others, as well as fear of contracting a new and more dangerous variant. For many, fear became a part of daily life:

It messes with your mental state, this COVID does… You’re going to stores and stuff and people [are] coughing and you [are] looking at them like, “Do you have COVID?”—*Black, Woman*

Participants described grief and trauma in their communities from being unable to comfort sick or dying family members and friends due to restrictions; the sheer number of deaths in the community; the lack of closure and comfort from having socially isolated or virtual funerals; and unexpectedly losing loved ones and acquaintances:

I have witnessed someone who was praying for me because I was sick, die from COVID. … It really traumatized me because I felt like this woman was healthy and here she is dying. She’s been praying for me and hoping for me. —*Black Woman*

Parents discussed the emotional impact of virtual schooling and social distancing on their children, who were also unable to experience the social interaction and milestones associated with attending in-person events:

We went out once to get supplies, and we were in the house for three straight months. Didn’t let nobody in. It has sent her into a depression, because special days like birthdays, we couldn’t do anything. So she went into a depression. She’s nine*.—Black Woman*

### Financial difficulties

Loss of income due to reduced work hours or loss of employment was among the most frequently described effects of the pandemic and the more immediate impacts felt by participants:

But we were out of work for a while. First they called me and then my husband… But I had to be providing for the house for like three months without him working. Then it was very difficult because he was always the one who contributed most of all the expenses. *—Latinx Woman*

Lockdowns and fear of exposure to the virus were the primary drivers of job loss. In one case, the participant had to choose between having an income and a place to stay:

*Then I stayed with some friends for a few months… It was fine to stay with them before, even though I was working at the grocery store, but now, my friend’s parents had to move closer to help take care of their kid… and since they’re older they’re vulnerable to COVID so I couldn’t work at the grocery store. So that’s why I just needed to quit in order to be able to live here. —Black Woman*

### Unwanted changes in lifestyle

Participants spoke at length about having to alter their lifestyles and adopt prevention strategies. They noted how hard it was to access key resources at first – including masks, supplies, and relevant information. Once they were able to access these resources, precautionary measures became a part of daily life:

We’ve all been a little bit more educated on it, and how to be safe, and getting used to certain routines… I’m still not doing my grocery shopping. I’m still having that delivered, just because we do also work directly with seniors. For their sake, also, we don’t want to expose them because some of them have decided they don’t want to get vaccinated as well. *—Latinx Woman*

Participants noted a few unintended effects from mitigation practices, such as difficulty breathing through masks. Of all the recommended guidelines, the most difficult by far was social distancing, due to its effect on their social life and mental health:

When you’re used to being around your family and stuff like that, it’s kind of hard to break away from that, from your everyday thing that you do. Like a person wakes up every day and hugs their mother, to wake up one day and you can’t hug your mother because of the COVID thing. That’s a very hard cycle to break. It just doesn’t come overnight. And it’s not easy. It is not easy at all. *—Black Man*

### Positive outcomes

Participants identified silver linings, demonstrating resilience despite experiencing hardship from the pandemic. They reported feeling more gratitude for life, benefits from remote work, and seeing greater solidarity among community members:

I had a sense of help, well, tremendous solidarity from neighbors, friends, church, community. Look, it is an impressive thing … I say, here in the United States, you can live somewhere 20 years and not know your neighbor, and I ended up, yes, meeting people and offering them help and vice versa. *—Latinx Woman*

Some individuals felt supported by the government and science through the availability of free COVID-19 testing, free vaccines, and unemployment support. The most common positive outcome was more time and bonding with loved ones:

It gave me better time to not only heal but get to know myself a little better, and get to know my daughter a lot better, and her getting to know her mother a lot better. So it was also a bonding thing, too. —*Black Woman*

## Perspectives on why the pandemic’s impact has been higher on Black and Latinx communities

Participants’ perspectives on the reasons for the disproportionate impact on their communities highlighted five sub-themes: (1) racism and systemic inequities in social determinants of health; (2) lack of access to information and language support; (3) cultural practices and lifestyles; (4) distrust in government and the medical system; and (5) individual responses to the pandemic.

### Racism and systemic inequities in social determinants of health

Participants overwhelmingly identified the role of systemic racism and associated disparities in social determinants of health as the primary reasons for the pandemic’s greater impact on their communities. One participant described this as follows:

Racism is systemic… and we don’t want to talk about how it affects communities of color and how it has affected this pandemic and that it’s insidious, so you don’t ever see it, but it’s there. It’s [the] underlying factor in a lot of things. Sometimes it’s so insidious that [it] is within the policies and the practices that we can’t even see and that’s even in public health. *—Black Woman*

Participants noted the higher prevalence of preexisting health conditions in Black and Latinx communities and provided examples of wide-ranging disparities extending to COVID-19. These examples broadly relate to three fundamental problems: lack of access to healthcare, lack of access to critical resources, and high risk for exposure to the virus.

They believed lack of access to healthcare led to a higher burden of COVID-19 in their communities in many ways. Latinx individuals in particular identified language barriers to accessing and fully benefiting from healthcare, and undocumented immigrants feared exposure due to their immigration status. Both Black and Latinx participants noted a reluctance in their community to seek help out of fear of experiencing discrimination, which delayed care in the early stages of illness. Participants talked about poverty and lack of health insurance as further causing delays in seeking medical care:

Another thing is that we also have no insurance … and then also people will not go for that any little thing, or some symptom, and neither did they go for fear of that too. *—Latinx Woman*

Participants also described systemic disparities that limit access to other critical health-related resources such as food, housing, and transportation. For example, a participant observed that grocery stores in certain areas were consistently out of supplies, forcing community members to go to other places even though transportation itself was a challenge. A few participants observed housing situations that make quarantining and isolation difficult, such as homeless individuals congregating at shelters. Others described reluctance among community members to apply for benefits because they feared racial discrimination or felt ashamed to ask for help:

Some people will not even go and apply for the SNAP benefits. They just, “No, I’m not going to apply.” Some people won’t go to the places to get the boxes of food. “No, I’m not going in that line and everything” because they drive up in the nice car and people say things. *—Black Woman*

Finally, many interviewees attributed the higher burden of the pandemic to an overrepresentation of community members among essential workers in restaurants, grocery stores, construction, and other industries; all jobs with high risk of exposure:

So number one is our work. I think that impacted a lot that these communities were the essential workers and in the beginning, their employers weren’t putting anything in place to make it safer or enforcing like, wear masks… They were all outside of the homes working. So it’s very few of the people that I know that were working from home that actually got it. *—Latinx Woman*

Participants often discussed this reason for higher pandemic burden alongside broader structural and systemic barriers in education and job opportunities, noting that Black and Latinx individuals in the U.S. are more likely to have low income and a greater need for work, especially among the immigrant population who are ineligible for governmental aid.

### Lack of access to information and language support

Participants discussed lack of language support, technical and social barriers to accessing information, as well as issues with misinformation and timeliness of information.

Latinx participants emphasized the need to have materials in their native language, as well as interpreters at medical appointments and vaccination sites for effective communication with healthcare providers:

I was telling [my doctor] that a lot of Latino people come into the clinic, and I asked him why they didn’t make any flyers in Spanish for the people who can’t understand the language, because there are families who can’t read, like myself, I can’t read English, but I can read Spanish. —*Latinx Woman*

Participants observed problems with using medical terminology – such as how many people used “isolation” and “quarantine” interchangeably – and the critical need for conveying information in ways that speak to the community:

You take a professionally developed flyer that was rubber stamped by organizations and you process it in community and there’s still a disconnect in approach, in language, in understanding, and so many other factors. *—Black Woman*

Individuals were also troubled about receiving information long after others had received it. Having limited access to phones and the internet made it difficult to share information among community members. Some people didn’t watch television or could not understand the information conveyed on channels broadcasting in English, relying instead on information from family and community members. Most people mistrusted and at times avoided information on social media:

Social media has really contributed to this misinformation. And I’m not saying that social media doesn’t at times present accurate or truthful information, but really the way I understand the way that [it] is constructed, it really is geared towards the appetite of the person receiving the social media that further validates or forms opinion that already exists with that individual. It doesn’t necessarily challenge or introduce new information to that individual as a way to contrast… *—Black Man*

### Cultural practices and lifestyles

Cultural practices and lifestyles were among the reasons participants gave for higher exposure to the virus in Black and Latinx communities. They noted that it’s more common in their communities to live in large intergenerational households with little if any room for isolation if needed:

I think most of us live in these, what’s the word, intergenerational communities. A lot of families do, so people are in close contact, so I think that makes one significant impact. *—Black Woman*

Both Black and Latinx participants observed their community and cultural norms as being more sociable, including frequent gatherings and closer interactions:

As Hispanics, [we] have a culture of friendship and our friendships are a little closer. When you greet people, you give them a hug; sometimes you do not even know them, and you give them a hug. It is part of our culture. *—Latinx Man*

### Distrust in government and the medical system

A majority of participants identified widespread distrust of government and the medical system as important reasons for the pandemic’s impact on their communities. Distrust affected how community members processed pandemic-related information and whether they were willing to follow public health guidelines:

Like anything the government says to do or anybody in power or whatever government official and everything, [people] are like, “They [are] telling us? I’m not going to do it, just because they’re telling me to do it. They can’t tell me what to do.” *—Black Woman*

Distrust of medical and government institutions was further exacerbated by the perceived speed at which the COVID-19 vaccines were developed, causing suspicions about recruitment into vaccine trials and concerns about getting vaccinated:

But what’s in [the vaccine]?.. I know y’all probably had an idea that a pandemic was going to happen because I’m like, “Y’all sure got this sucker together fast, all these other diseases and things that [are] out here and you can’t find the cure for it.” But with COVID-19, there you go, we got a vaccine. *—Black Woman*

### Individual responses to the pandemic

Participants observed that individual responses to the pandemic varied within their community and expressed disappointment that some members of their community did not take the pandemic seriously. Examples included not following mitigation practices and engaging in risky behaviors like traveling. Due to general mistrust and politicization, some people worried about being stigmatized for following recommended guidelines:

I don’t think the wash your hands or mask-wearing, unless there is an underlying health condition, is problematic… but the issue is going to be, if I feel stigmatized in wearing a mask, that’s the issue that’s going to present itself. Right? That goes along the lines of [de]humanizing a certain group, right? *—Black Man*

Participants attributed the perceived lack of appropriate responses to the pandemic to a lack of information or the misinformation propagated by social media. They also noted that some individuals assessed their personal risk as low and did not want to change their lifestyles – a response that was observed more frequently in younger members of the community:

I think that some folks didn’t take it serious, that’s one. Even I have children that range from age 11 to age 31 almost… Some of my children and even their friends were just, “we’re just living our life and we don’t care about COVID.” *—Black Woman*

## Strategies used to alleviate the impact of COVID-19

To alleviate the pandemic’s burden on their personal lives, participants worked at strengthening bonds with loved ones in as many ways as they could: enhancing relationships virtually, decompressing through conversation, and finding safe ways to celebrate important moments. Some turned to places of worship, prayer, or meditation.

To avoid getting overwhelmed by the constant stream of pandemic information, varied opinions, and misinformation, some participants chose to minimize their time on social media. To circumvent the problem of distrust in government and medical professionals, they turned to more trusted information sources, especially community members with lived experience of COVID-19:

Well, I trust a lot of information that comes from people. I want to hear information from people who survived it, people who lived through it, that struggle with it and that overcame the infection or the virus. *—Black Man*

Participants trusted other Black and Latinx individuals in the community, including community leaders and faith leaders:

And so you have places of worship that should, I believe first, I mean, first and foremost, be amongst the leaders in communicating information that’s accurate to the community, right? *—Black Man*

## Discussion

The interview findings reflect the experiences and perspectives of Black and Latinx individuals in four Michigan counties during a critical period in the pandemic. At the time of the first interviews, COVID-19 vaccines were just becoming available to medical professionals, and the Michigan Coronavirus Racial Disparities Task Force appointed by the state government had just released an interim report showing the disproportionate burden on Black and Latinx communities [28]. As the study unfolded and the pandemic evolved, we were able to document the emerging struggles, questions, and hesitancies of community members. These perspectives provide important insights for developing crisis response strategies and public health messaging that acknowledge the concerns and cultural needs of racial and ethnic minority populations. Preparation based on lessons learned from the COVID-19 pandemic is essential for effectively and rapidly responding to crisis situations in the future.

Participants in this study attributed the higher burden of the pandemic on their communities to many underlying causes. Findings from our study in Michigan are consistent with other research from around the United States addressing the reasons for a greater impact of COVID-19 on Black and Latinx communities [34,35]. Participants emphasize similar factors including health and socioeconomic disparities deeply rooted in systemic biases and racism [36]. Barriers to accessing healthcare and critical resources, as well as financial strain and lack of job security in unstable work situations, place these communities at a risk for poor health outcomes [37–39]. Mistrust in institutions spurred by historical mistreatment, causes more individuals to respond skeptically to recommended prevention and mitigation strategies [40]. At the same time, recommended strategies are rarely tailored to cultural needs [41].

Our participants vividly described the effects of these race-based and socioeconomic disparities on their lived experience in terms of worsened health as well as socially and financially upended lives. Negative effects on mental health were particularly prevalent. These findings highlight the pandemic’s overwhelming impact on health in communities with a history of poorer health outcomes [42].

Poor health and social outcomes from deeply ingrained and widespread racial and ethnic disparities have led to a general sense of frustration. Black and Latinx participants in this study indicated that their communities have had neither the access to resources nor the timely and accurate information they needed to adequately understand and effectively respond to the situation unfolding around them. These experiences perpetuate the cycle of mistrust that is bound to affect responses in future health crises [43,44]. This study provides evidence for that: Participants described how mistrust in institutions of the government and the medical system, stemming from historical mistreatment as well as immigration policies, caused Black and Latinx community members to delay healthcare, ignore mitigation practices, and shun vaccination. The compounding effects of multiple crises are a major public health threat that cannot be ignored.

## Recommendations

Breaking the cycle of mistrust will take a long time, but should start with genuine, clear, and transparent communication that engages Black and Latinx communities and acknowledges their past and current experiences with racial and socioeconomic disparities. In public health crises, it is critical for elected officials and the medical and scientific communities to acknowledge the situation and provide tangible support. Our participants noted the offering of free COVID-19 testing and free vaccines by the government and health experts among positive experiences. However, our findings show that it is equally important to create local opportunities to hear from both community members with lived experience and community leaders. Participants expressed trust in community and in individuals with experiences related to being disenfranchized or having COVID-19. As in other communities across the United States, in Michigan, we saw that community members including faith leaders can serve as trusted messengers for Black and Latinx residents, and can help them make more informed decisions about their health [45,46].

It is essential to acknowledge trusted information sources and to ensure that those sources have the necessary and accurate information to communicate to the public. Therefore, a truly community-engaged approach involving authentic, long-term partnerships between community leaders, community organizations, health experts, and government is crucial. These relationships must be established and nurtured on an ongoing basis, not spontaneously pursued in crisis situations. Such partnerships can learn from experiences where similar power dynamics exist, including established principles and best practices in community-based participatory research [33]. These approaches recognize and elevate community perspectives and community expertise in equal partnership with academic collaborators (i.e., domain knowledge experts). Institutions can also formally engage and empower community members to help identify and eliminate institutional practices and policies that sustain and propagate disparities and mistrust.

Our findings also highlight the importance of leveraging both cultural humility and cultural competency to effect action [47]. For instance, Black and Latinx individuals are more likely to live in multigenerational homes, with elderly parents, grandparents, and extended family. In the context of COVID-19, this made social distancing harder, placing other family members, especially those who are elderly or immunocompromised, at higher risk of infection [48]. In this context, while communicating scientific facts such as the need to self-isolate is necessary, it is not sufficient; we must seek to learn and incorporate cultural and community-specific approaches to identify potential barriers when attempting to implement prevention measures and offer appropriate solutions. For example, media campaigns, which can include appearances on television or radio by community leaders and volunteer groups, can adapt public health messaging to specific cultural contexts. If done well, they can serve to reinforce a sense of community while also establishing preventive strategies and positive health practices [49]. In this study, we saw that solidarity within the community and positive relationships with family and friends are important for Black and Latinx community members and serve as critical supports.

Participants in this study talked about widespread systemic disparities and getting accessible information and resources later than others. This situation existed before, but the pandemic magnified it further. When disparities are starkly evident even outside of crisis situations and are identified as the reasons for the greater impacts of crisis situations, emphasizing specific measures such as vaccines as the means for protection runs the risk of appearing hypocritical and perpetuating distrust. Community members want to feel cared about – not only during crisis situations – as opposed to feeling they are being made to do things. To provide creative solutions that are more effective in the long term, public health campaigns must focus on individual, family, and community wellbeing – which includes but is more than vaccination – allowing people to feel they are heard and cared for. Communicating these messages in accessible ways that resonate with community members is essential. We believe this can be accomplished more effectively through collaboration between community and domain experts.

Following the expiration of the federal Public Health Emergency for COVID-19 in the United States on May 11, 2023, prevention and mitigation strategies were significantly relaxed and life has mostly returned to pre-pandemic routines [50]. However, we must not return to pre-pandemic norms regarding public health more broadly. As the Michigan Coronavirus Racial Disparities Task Force recommended, we must continue to document and publicly report racial disparities in COVID-19 and social determinants of health [51]. We must also increase our efforts to support communities in alleviating health conditions and social needs that were exacerbated by the pandemic. Spreading awareness of ongoing needs, building capacity to help communities become more resilient, and sustaining effective mechanisms for reducing the impact of racial disparities will be crucial.

For participants in this study, one of the greatest impacts of the pandemic was on mental health. This is consistent with research focusing on the United States population that demonstrates racial and ethnic disparities not only in how the pandemic influenced mental health but also in mental health care [42,52]. As Thomeer and colleagues discuss, for Black communities the effects of the pandemic coincided with other sources of racial trauma such as the murder of George Floyd, while in Latinx communities, factors ranging from high levels of deportation and family separation to discrimination and political rhetoric caused sustained psychological harm. Given the disparities in accessing mental health care, it is essential to identify ways of empowering communities to build capacity for healing and resilience. One example of this is supporting Mental Health First Aid trainings, as offered by some county community mental health agencies in Michigan, that allow community members to develop skills to help others experiencing mental health conditions [53]. Another example is developing and supporting a sustainable workforce of community-based, trusted providers such as community health workers (CHWs) to serve as linkages between health care and community settings for individuals with health and social needs [54]. For instance, we believe recent policies regarding Medicaid reimbursements for CHW services represent examples in the right direction [55]. Mobilizing community members, community leaders, and health workers through such initiatives will promote leadership and allow for community-oriented and culturally sensitive care [56,57].

Similarly, we must also support community-based organizations focused on services addressing social determinants of health needs, such as housing, food, and transportation, who can advocate for the community. The health of communities and their ability to face future crises is unlikely to be sufficiently strengthened by placing resources disproportionately into traditional healthcare settings. The COVID-19 pandemic shone a global spotlight on entities working to alleviate social needs that contribute to poorer health outcomes. These entities are in high demand but have limited resources. This is an area of potential impact for policymakers. Organizations that provide housing services (which worked to limit exposure and spread of the virus during the pandemic), food, and transportation – among others – should have the resources necessary to meet community needs. It is essential to find ways to increase and sustain the capacity of these organizations.

## Study Limitations

Because our study is based on qualitative interview data, the findings are specific to the study context and highlight the experiences of a specific population at a particular time. The interviews were conducted during a peak period in the pandemic, from December 2020 to June 2021, when the situation was rapidly evolving. As a result, participants’ perspectives may not reflect their pandemic experience as a whole, but rather a specific moment in time. Finally, individuals between the ages of 18–29 were significantly underrepresented in our interviews, as only two participants fell within this age range. Other studies are necessary to see whether the results are consistent with experiences across populations. Despite these limitations, our findings are consistent with Black and Latinx experiences others have observed across the United States [4,7,10].

## Conclusion

We used a community-based participatory research approach to understand the lived experiences of Black and Latinx community members during a peak period of COVID-19. The MICEAL Steering Committee was instrumental in providing direction and insights in all aspects of the study; for example, development of the interview protocol and interpretation of the findings. Our interviewees described how they faced disproportionate burdens during the pandemic. The findings reflect community members’ needs and the discussions and narratives that *they* felt were important to highlight. Health inequities raise key issues that should be addressed, regardless of whether a pandemic situation exists. While effective leadership is needed to provide support in times of distress and dispel misinformation, it is equally crucial to work toward dismantling racial and socioeconomic disparities during normal times, so that individuals feel supported, secure, and more trusting of public health officials during times of crisis.
